# The utility of simultaneous CT-guided localization for multiple pulmonary nodules using microcoil before video-assisted thoracic surgery

**DOI:** 10.1186/s12890-021-01393-x

**Published:** 2021-01-25

**Authors:** Yanyan Xu, Lingchuan Ma, Hongliang Sun, Zhenguo Huang, Zhenrong Zhang, Fei Xiao, Qianli Ma, Jie Lin, Sheng Xie

**Affiliations:** 1 Department of Radiology, China-Japan Friendship Hospital, No. 2 Yinghua East Street, Chaoyang District, Beijing, 100029 China; 2 Department of Radiology, The People’s Hospital of Wenshan Prefecture, Wenshan, 663099 China; 3 Department of Thoracic Surgery, China-Japan Friendship Hospital, No. 2 Yinghua East Street, Chaoyang District, Beijing, 100029 China; 4 Department of Pathology, China-Japan Friendship Hospital, No. 2 Yinghua East Street, Chaoyang District, Beijing, 100029 China

**Keywords:** Pulmonary nodule, Localization, Video-assisted thoracoscopic surgery

## Abstract

**Background:**

To evaluate the feasibility and safety of microcoil in simultaneous localization for multiple pulmonary nodules before video-assisted thoracic surgery (VATS).

**Methods:**

Twenty-eight consecutive patients (26 two-nodule, 2 three-nodule; 58 nodules in total; Group A) underwent simultaneous CT-guided localization of multiple pulmonary nodules before VATS using microcoil. Successful targeting, localization, and VATS were defined as implantation of microcoil at the target site on CT image which was obtained immediately after the marking procedure, visualization of nodule location, and complete resection of the target nodule with adequate margin, respectively. Meanwhile, the clinical characteristics, localization procedure-related variables of the nodules and procedure-related complications in group A were also assessed and compared with those in a control group (221 single-localization procedures in 221 patients; Group B).

**Results:**

Similar rates of successful targeting, localization and VATS were observed in group A and B (96.6% vs. 98.2%; 91.4% vs. 91.0%; 100% vs. 99.1%). Although the rate of overall complications (including localized pneumothorax and intrapulmonary hemorrhage) was a bit higher in group A than that in group B (32.8% vs. 30.8%, *p* = 0.771), only minor complications were observed in the subjects of the two groups with no need for further treatment. In addition, the duration of simultaneous localization procedures was significantly longer than that of single localization (24 ± 7.5 vs.13 ± 6 min, *p* < 0.001).

**Conclusions:**

CT-guided simultaneous microcoil localization for multiple pulmonary nodules before VATS was clinically feasible and safe with acceptable increase in the procedure time. Compared with localization for a single pulmonary nodule, simultaneous microcoil localizations for multiple nodules were prone to pneumothorax and hemorrhage. However, no statistically significant differences were observed between the two groups.

## Background

Owing to the worldwide concerns about lung cancer screening and the improvement of imaging modalities, the detection of small pulmonary nodules is increasing, especially the rate of simultaneous multiple nodules in a single patient [1, 2]. In a study conducted by Marjolein et al. [3], the baseline nodule count (the number of nodules per screen) was not associated with the probability of lung cancer and each nodule should be assessed separately.

For indeterminate nodules, excision biopsy, which removes the entire nodule at one setting, is an available solution either for patients or physicians, especially for anxious patients [4]. Preoperative tumour localization can greatly improve the accuracy of resection and decrease the rate of conversion to thoracotomy [5–14]. CT-guided microcoil localization of pulmonary nodule before video-assisted thoracic surgery (VATS) has been considered as a safe and accurate technique with a high success rate and low complication rate [5–14]. However, considering the procedure time, possible changes in body position, potential morbidity and complications, it is still a challenge to perform simultaneous localization of multiple nodules in one procedure.

Although the simultaneous localization has been performed or mentioned in prior studies [15–19], few studies have focused on the microcoil utilization in simultaneous localization before VATS [19]. Thus, we retrospectively summarized the experiences of CT-guided microcoil localization in lung nodules and evaluated the efficacy and safety of microcoil utility in simultaneous localization.

## Methods

The retrospective study was approved by the Ethical and Scientific Committees of our hospital, and informed consent was waived. Informed consent for CT-guided percutaneous localization was obtained from each patient before the procedure was performed.

### Study subjects

From June 2016 to March 2019, 249 consecutive patients (90 males, 159 females) with 279 pulmonary nodules underwent CT-guided microcoil localization prior to VATS at our radiology department were enrolled into this study. As the same patients were previously analyzed with a different objective [20], the detailed information regarding the exclusion criteria for CT-guided microcoil localization is described in the Additional file 1.

Of these, 28 patients (male/female: 10/18; mean age: 57.7 ± 8.0 years) underwent simultaneous as above multiple pulmonary nodules (26 two-nodule, 2 three-nodule, 58 nodules in total; Group A), and the remaining 221 patients (male/female: 80/141; mean age: 57.3 ± 11.3 years) underwent localization procedures for 221 nodules (Group B). The clinical characteristics of the patients were summarized in Table 1.

**Table 1:** The clinical information of the patients underwent CT-guided microcoil localization

Variable	Different groups		*p*-value
Patients No	28	221	–
Nodule No	58	221	–
*Age (y)*			
Mean ± SD(range)	57.7 ± 8.0 (39–77)	57.3 ± 11.3 (26–81)	0.825
≤ 55/ > 55	13/15	94/127	0.695
*Gender*			
Male/Female	10/18	80/141	0.960
*Smoking status*			
Ex- or current/Never	2/26	28/193	0.546
*Cancer history*			
Primary lung/Extra-pulmonary/No	0/2/26	10/18/193	–
*Previous thoracic operation*			
Yes/No	0/28	10/211	–
FEV_1.0_/FVC (%)			
Mean ± SD (range)	74.3 ± 9.9 (44.6–91.5)	77.2 ± 7.4 (52.9–91.5)	0.061
*Localization procedure time (min)*			
Median ± IQR (range)	24 ± 7.5(13–45)	13 ± 6(5–52)	< 0.001
*The time to the operation (hour)*			
Median ± IQR (range)	19.2 ± 16.9 (0.9–72.6)	20.0 ± 12.7 (1.0–95.7)	0.202
≤ 24/> 24	21/7	144/77	0.299

Abbreviation: FEV_1.0_/FVC, forced expiratory volume at 1 s/forced vital capacity; SD, standard deviation; IQR, interquartile range

### Procedures

All the planning and localizing CT scans were carried out using 16 detector-row scanner (Aquilion 16; Canon Medical Systems, Japan). The detailed information regarding the CT scanning and CT-guided localization procedures is described in the Additional file 1. And the procedure-related complications were evaluated based on the Society of Interventional Radiology Standards of Practice Committee Classification [12].

**Fig. 1:**
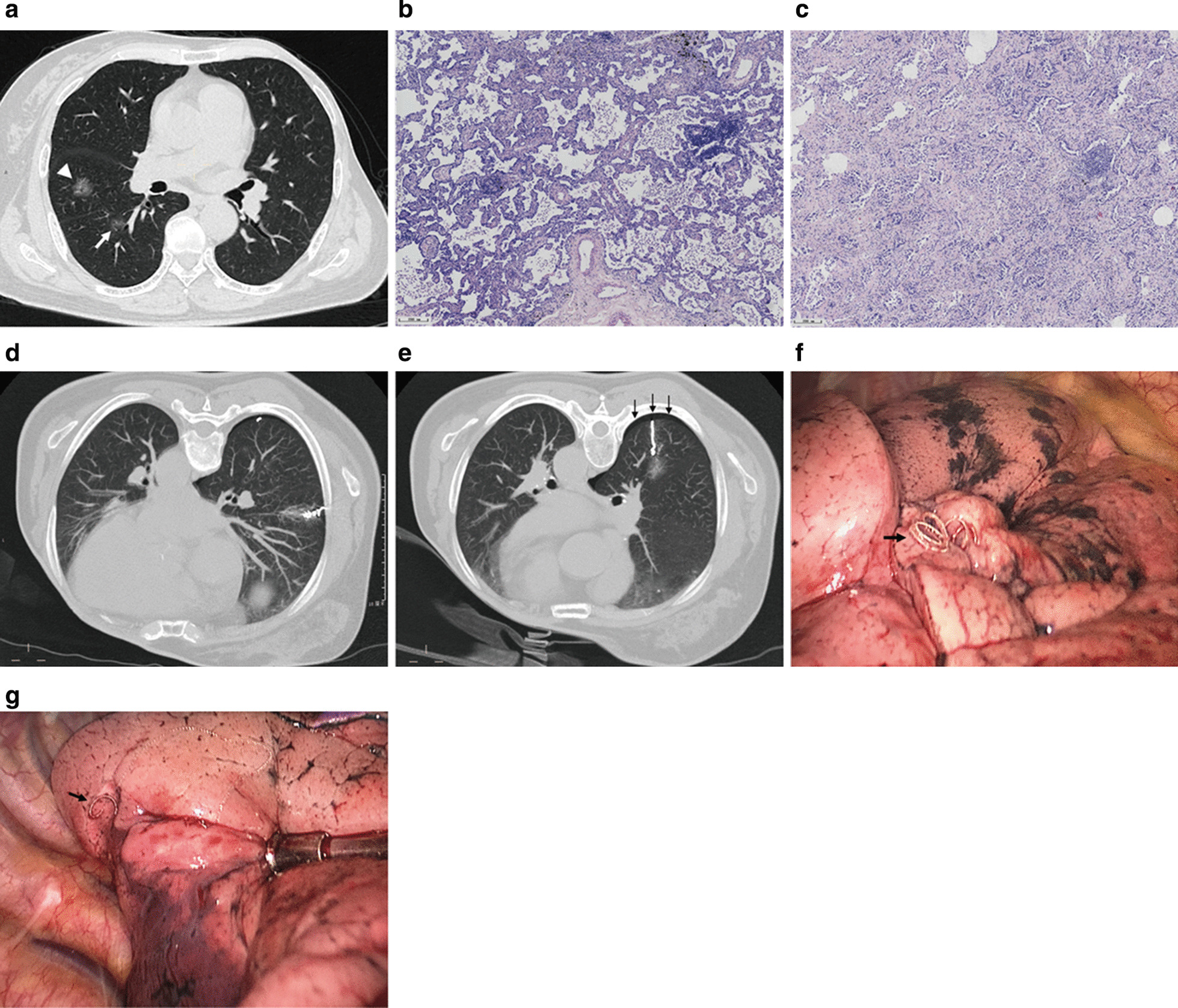
In a middle-aged patient, two ground-glass opacity nodules were located in the lateral segment of the right middle lobe (**a** white arrowhead; axial section size was about 1.4cmx1.5 cm) and the dorsal segment of the right lower lobe (**a** white arrow; axial section size was about 0.9cmx1.1 cm), respectively. Histopathologically, the lesion in the right middle lobe was diagnosed as minimally invasive adenocarcinoma with predominant lepidic growth (**b** H&E, × 50;) and the one in the lower lobe was invasive adenocarcinoma presented as acinar pattern (**c** H&E, × 50) (Digital slice scanning system: PRECICE 500B; UNIC TECHNOLOGIES INC, Beijing, China). Neither of them is metastatic disease. The patient maintained a prone position during the interventional procedure (**d** middle lobe implantation; **e** lower lobe implantation) and the entire implantation was successfully performed with minor pneumothorax (**e** indicated with black arrows). The tails of the microcoils were visualized during VATS (indicated with black arrows; **f** corresponded to **d**; **g** corresponded to **e**)

The excision of the complete microcoil and nodule was carefully performed with the guidance of VATS and preoperative localization CT (Fig. 1a–g), and the specimen was immediately sent for frozen section to assess whether the lesion was completely resected and/or the surgical margin was enough. Complete lobectomy was performed unless the lesion was found to be noninvasive cancer, or the patient had an inadequate cardiopulmonary reserve, or the patient had lung resection before, or the patient declined to extended resection.

### Assessment

The successful targeting rate, localization rate, VATS rate, and procedure-related complications rate were calculated based on the total number of the nodules [11, 21]. Successful targeting was defined as implantation of microcoil at the target site adjacent to a nodule on CT image which was obtained immediately after the marking procedure and the rate was calculated as follows: (number of successful targeting procedures/number of all localization procedures in each group) × 100; successful localization was defined as detection of nodule location and the rate was calculated as follows: ([number of successful targeting procedures-number of dislodgements or misses under the thoracoscope]/number of all localization procedures in each group) × 100; successful VATS was defined as a complete resection of the target nodule with adequate margin and the rate was calculated as follows: ([number of successful VATS /number of all localization procedures in each group) × 100. The severity of procedural complications was also recorded according to the Society of Interventional Radiology Standards of Practice Committee classification of complications [12].

In addition, all preoperative CT data was transmitted to the picture archiving and communication system (PACS) and scanner workstation. The nodule characteristics and localization procedure-related variables, including nodule location, size, type, depth from pleura, presence of emphysema, patient position for localization procedure, needle-pleural angle, pleura-microcoil distance, presence of “pleural indentation”, scapulae-covered sign, localization procedure time as well as the time to the operation were measured and recorded. As the aforementioned variables were previously analyzed with a different objective [20], The detailed definition or description is described in the Additional file 1. The comparison of clinical characteristics and procedure-related variables between Group A and B were recorded in Tables 1 and 2.

**Table 2:** Summary of nodule characteristics

Variable	Different groups		*p*-value
	Group A	Group B	
*Nodule size (mm)*			
Median ± IQR (range)	7.1 ± 3.6 (2.3–18.9)	9.8 ± 5.8 (2.8–26.8)	< 0.001
≤ 5 mm	10	12	< 0.001
> 5–10 mm	38	106	
> 10–15 mm	9	74	
> 15 mm	1	29	
*Nodule location*			
Right/Left side	39/19	131/90	0.268
RUL/RML/RLL	21/0/18	86/14/31	0.011
LUL/LLL	10/9	63/27	
*Nodule type*			
Solid/Part-solid/GGO	20/12/26	36/33/152	0.002
*Depth from pleura (mm)*			
Median ± IQR (range)	12.6 ± 13.6 (0.0–44.4)	10.3 ± 16.7 (0.0–53.1)	0.781
≤ 5 mm	14	52	0.159
> 5–10 mm	12	57	
> 10–15 mm	13	25	
> 15 mm	19	87	
*Presence of emphysema*^*★*^			
Yes/No	4/54	6/215	0.224
*Position for localization procedure*			
Supine/Prone	24/34	108/113	0.309
*Needle-pleura angle (°)*			
Mean ± SD(range)	64.0 ± 14.0 (32.4–88.9)	61.2 ± 15.7 (21.1–89.8)	0.225
≤ 30°	0	8	0.410
> 30–60°	23	93	
> 60°	35	120	
*Pleura-microcoil distance*^*▼*^*(mm)*			
Mean ± IQR (range)	35.2 ± 9.4 (4.0–57.8)	34.07 + 10.3 (1.0–55.9)	0.378
≤ 10 mm	2	11	0.211
> 10–20 mm	0	13	
> 20–30 mm	11	43	
> 30 mm	45	154	
*Presence of pleural indentation*			
Yes/No	10/48	60/161	0.121
*Presence of the scapulae-covered*			
Yes/No	4/54	42/179	0.027
*Procedure-related complications*			
Yes/No	19/39	68/153	0.771
*Pneumothorax*			
Yes/No	16/42	67/154	0.686
*Intrapulmonary haemorrhage*			
Yes/No	7/51	26/195	0.949
*Surgical procedure*			
Wedge resection/Anatomic resection^●^/Open thoracotomy	46/12/0	134/85/2	**-**
*Pathologic result*^*▼▼*^			
Primary lung cancer/metastasis/Benign lesion	37/0/21	175/3/43	**-**

Abbreviation: IQR, interquartile range; SD, standard deviation; GGO, ground glass opacity; RUL, right upper lobe; RML, right middle lobe; RLL, right lower lobe; LUL, left upper lobe; LLL, left lower lobe

^★^The emphysema region was around the needle insertion pathway

^▼^ Pleura-microcoil distance was measured along the needle insertion pathway

^●^Anatomic resection included lobectomy and segmentectomy

^▼▼^Detailed pathologic results were summarized in the Table 3

### Statistical analysis

Statistical analysis was performed using SPSS 17.0 software (SPSS 17.0 for Windows, Chicago, IL). The Kolmogorov–Smirnov test for normality was performed on continuous variables and the graphical spread of the data was visually inspected. Descriptive statistics were shown as mean ± standard deviation (SD) or median ± interquartile range (IQR) for continuous variables, and as frequency and percentage for categorical variables.

The comparison of clinical characteristics and microcoil localization procedure-related variables between group A and group B were analyzed by Independent-samples *t*-test/Mann–Whitney U test, and the chi-square test/Fisher exact test. A two-sided *p*-value less than 0.05 was considered statistically significant.

## Results

### The successful targeting, localization, and VATS rate for group A and B

For group A (multiple nodules per person), according to the CT scan obtained immediately after the marking procedure, two microcoils which were implanted on the pleural surface and did not reach the target parenchymal regions (one of the nodules was performed in early-stage without repeated puncture, the other was given repeated puncture), thus the successful targeting rate was 96.6% (56/58).Three microcoils dislodged into the thoracic cavity after initial deflation of the lung, thus the successful localization rate was 91.4% (53/58).

For group B (single nodule per person), four microcoils did not reach the target parenchymal region on CT scan and repeated punctures were performed immediately, thus the initial successful targeting rate was 98.2% (217/221). 12 microcoils dislodged into the thoracic cavity, 3 adhered to the parietal pleural and 1 fell on the diaphragmatic surface when the target lung collapsed, thus the successful localization rate was 91.0% (201/221).

For most of the nodules with failed targeting or localization mentioned above, subpleural haemorrhage or puckering of the visceral pleura combined with intraoperative palpation served as additional guidance for successful resection. Only two patients in group B were converted to open thoracotomy, one due to diffuse pleural adhesion, the other for inability to perform bronchoplasty after nodule resection with a video-assisted thoracoscope. The VATS success rate of group A and B was 100%, 99.1%, respectively.

### Localization procedure time and procedure-related complication rates

Although the nodules in the patients of group A were all in the unilateral lung distribution, twelve patients (12/28; 42.9%) have changed body positions during interventional procedures. The procedure time of group A was significantly longer than that of group B (24 ± 7.5 vs. 13 ± 6 min, *p* < 0.001).

With respect to procedure-related complications, only minor complications (including localized pneumothorax and intrapulmonary haemorrhage; class A/B) were observed in the subjects of the two groups with no need for further treatment. Furthermore, there was no significant difference in the rates of overall procedure-related complications between the two groups (32.8% vs. 30.8%, *p* = 0.771).

### Pathological result

The detailed pathological results for the nodules were summarized in Table 3. Over half of the nodules in both groups were confirmed as malignant lesions (63.8% vs. 80.1%). The pathological results of multiple nodules in one same patient were inconsistent, which was revealed in half of the patients with multiple nodules (14/28).

**Table 3:** Summary of pathology in two groups

Pathologic process	Incidence	
	Group A (n = 58)	Group B (n = 221)
*Malignant*		
Invasive adenocarcinoma	16 (27.6%)	104 (47.1%)
Minimally invasive adenocarcinoma	15 (25.9%)	58 (26.2%)
Adenocarcinoma in situ	3 (5.2%)	10 (4.5%)
Squamous cell carcinoma	1 (1.7%)	1(0.5%)
Metastases	0	3 (1.4%)
Pulmonary carcinoid	2 (3.4%)	1(0.5%)
*Benign*		
Atypical adenomatous hyperplasia	6 (10.3%)	10 (4.5%)
Minute pulmonary meningothelial-like *nodules*	0	2 (0.9%)
Nodular lymphoid hyperplasia	3 (5.2%)	4 (1.8%)
Benign intraparenchymal lymph node	2 (3.4%)	4 (1.8%)
Localized pneumonitis	3 (5.2%)	13 (5.9%)
Cryptococcosis	2 (3.4%)	3 (1.4%)
Tuberculosis	0	2(0.9%)
Granulomas	2 (3.4%)	1(0.5%)
Sclerosing hemangioma	0	1 (0.5%)
Fibrotic nodule	3 (5.2%)	4 (1.8%)

## Discussion

Results of the current study indicate that preoperative microcoil localization technology is a safe and effective method for simultaneous multiple nodules as well as a single pulmonary nodule, with similar successful targeting (96.6% vs. 98.2%) and localization rates (91.4% vs. 91.0%). Meanwhile, the rates of overall procedure-related complications showed no statistically significant differences between the two groups (32.8% vs. 30.8%, *p* = 0.771). The high success rate of final VATS further confirmed the feasibility of simultaneous microcoil preoperative localization of multiple nodules.

Actually, with respect to simultaneous localization of multiple pulmonary nodules, the potential challenges and problems have been well described in previous studies [15–19], however, the authors adopted a hook-wire localization technique instead. The efficacy and safety of different localization techniques (including hook-wire, microcoil and lipiodol) for pulmonary nodules have been discussed and compared. Even though targeting success rates were similar amongst them, microcoil localization yielded the lowest complication rate [11]. Compared to single localization, simultaneously targeting multiple nodules equates to repetitive procedures in a short time, which is not only associated with potential increased risk of failure and complication but also challenging for both operators and patients.

An integrated and ideal process of microcoil localization in our interventional unit is “one line with two dots”, which means two ends of the microcoil are tightly deployed in lung parenchyma and the pleural surface, respectively. In our opinion, the main reason for targeting failure may be attributed to the insufficient depth of insertion. The pleura tented towards nodule instead of clean penetration by puncture needle (as evidenced by pleural indentation). In such cases, the needle tip appears close to the nodule on imaging but has actually not reached the desired region. In other words, it is targeting failure. The flexibility of microcoil makes it easy to curl and retract once it is ejected from out of the puncture needle. If there is no enough distance between the two ends of the microcoil to resist its own elastic recoil force, it would retract into the parenchymal or deploy on the pleural surface. And in the latter situation, microcoil often dislodged from the pleural when the targeted lung collapsed, which is localization failure. In the current study, either” presence of pleural indentation” or, “pleura-microcoil distance” showed no significant differences between group A and B. Therefore, it is reasonable to observe similar success rates of targeting and localization in two groups (96.6% vs. 98.2%; 91.4% vs. 91.0%).

Smaller nodule size and lower GGO rate were observed in group A (multiple nodules group). However, microcoil was implanted adjacent to lesion rather than through it, nodule size or type seemed to rarely influence the progress of the procedure. Although it was suggested that priority should be given to the most clinically significant nodule (such as the lesion difficult to locate or with malignant features) [15], we did not make such selection for puncture order when simultaneous localizations were performed. Although the rate of overall procedure-related complications was slightly higher in group A than that in group B (32.8% vs. 30.8%, *p* = 0.771), only minor complications [12] (including localized pneumothorax and intrapulmonary haemorrhage; class A/B) were developed in the two groups. There are two inevitable factors associated with the higher rate of complications in multiple nodules group: (1) multiple punctures in the ipsilateral lung [22]; (2) patient’s body position changes during the procedure. Hence, to some degree, the complication rate varied within a reasonable range. In addition, microcoil’s flexibility flexible but firm quality avoids the occurrence of parenchyma tear and its thrombogenic fiber coating reduces the risk of haemorrhage [13]. Therefore, in our opinion, it is a safe approach for simultaneous localization of multiple nodules.

Considering the nodule number and possible position changes needed during the procedures, it is not hard to understand that the duration of the localization procedures was longer for multiple nodules localization (24 ± 7.5 vs. 13 ± 6 min, *p* < 0.001). However, the procedure time could be greatly reduced in unchanging patient positions (only 13 min needed in two patients with two-nodules each) during the process, and similar results were observed in Iguchi et al. [15] study with hook wire system. On the other hand, even for a single nodule, the duration of the procedure would be delayed for variable factors, such as the shield of bone tissue nearby, irregular breathing pattern of patients. A much higher incidence of “the scapulae-covered” was observed in group B (42/221; 19.0%), resulting in 5 patients with a procedure time exceeding half an hour.

In addition, high rates of malignancy were demonstrated in both groups (63.8% [37/58] *vs* 80.5% [178/221]). Actually, most of the nodules were recommended for surgical treatment after the initial screening and follow-up period, which might account for the high malignant rate. Interestingly, it was observed that multiple nodules in the same patient showed inconsistent pathological results, and the ratio of such patients was up to 50% (14/28) in group A, which in turn emphasized the importance of separate assessment for lung nodules and necessity for simultaneous localization.

There are several limitations to the current study. First, it is a retrospective study that is limited to a single center. Second, the total number of patients with multiple nodules was relatively small. Third, there is a potential selection bias in the choice of patients with multiple nodules localization. Even though certain patients with multiple nodules would opt to have the most suspicious nodules localized rather than simultaneous localization for various reasons, we have not encountered that yet in our center. In other words, all the patients with multiple nodules we encountered selected simultaneous localization and radical resection. Fourth, occasionally, the proximal end of the microcoil detached from the visceral pleural surface and curled into the lung parenchyma. However, subpleural haemorrhage or puckering of the visceral pleura combined with intraoperative palpation served as additional guidance for successful resection. The results for those part of nodules were counted as successful localization in this study. Finally, only one localization technique was used in the study without comparison with other targeting techniques [11], such as hook wire localization, percutaneous injection of dyes, or intraoperative imaging.

## Conclusions

In conclusion, CT-guided simultaneous microcoil localization for multiple pulmonary nodules before VATS was clinically feasible and safe with an acceptable increase in procedure time. Compared with localization for a single pulmonary nodule, simultaneous microcoil localizations for multiple nodules has a higher incidence of pneumothorax and hemorrhage. However, no statistically significant differences were observed between the two groups.

## Supplementary information

**Additional file 1.** Detailed supplementary material for the following parts of Methods: Subjects, Procedures (CT-guided microcoil localization), Assessment.

## Data Availability

The datasets used and/or analyzed during the current study are available from the corresponding author on reasonable request.

## Abbreviations

VATS: Video-assisted thoracoscopic surgery
FEV_1.0_/FVC: Forced expiratory volume at 1 s/forced vital capacity
OR: Odds ratio
SD: Standard deviation
IQR: Interquartile range
PACS: Picture archiving and communication system
GGO: Ground glass opacity
WHO: World Health Organization
IASLC: The International Association for the Study of Lung Cancer;
ATS: American Thoracic Society
ERS: European respiratory society
RUL: Right upper lobe
RML: Right middle lobe
RLL: Right lower lobe
LUL: Left upper lobe
LLL: Left lower lobe
CI: Confidence interval
Ref.: Reference value
