# Supplementary material for: The utility of simultaneous CT-guided localization for multiple pulmonary nodules using microcoil before video-assisted thoracic surgery
Source: BMC Pulm Med. 2021 Jan 25;21:39. doi: 10.1186/s12890-021-01393-x (PMC7831238; doi:10.1186/s12890-021-01393-x)
Supplement: Supplementary file 1 — Additional file 1. Detailed supplementary material for the following parts of Methods: Subjects, Procedures (CT-guided microcoil localization), Assessment. [file 12890_2021_1393_MOESM1_ESM.docx]

**Supplementary material**

**Subjects**

Exclusion criteria for CT-guided microcoil localization were the following: 1) nodules adjacent to the hilum or apparent vascular structure; 2) lesions located in the bronchi; 3) patients who refused microcoil localization or VATS resection.

**CT-guided microcoil localization**

Before CT-guided localization procedures, patients were trained to hold the breath for 5-10 seconds at the end of inspiratory. All the planning and localization CT scans were carried out using the same 16-detector-row scanner (Aquilion 16；Canon Medical Systems, Japan). The following parameters for the planning CT: scanning method= helical acquisition mode; tube currents= 50mAs; tube voltage= 120kVp; rotation time= 0.5 sec; imaging FOV= 400; slice thickness= 5mm, the following parameters for the localization CT: scanning method= axial acquisition mode; tube currents= 50mAs; tube voltage= 120kVp; rotation time= 0.5 sec; imaging FOV= 400; slice thickness= 4mm. The limited slices were scanning including lesion and microcoil for saving radiation dose.

A planning CT scan was performed before percutaneous needle insertion. An appropriate puncture point on a patient’s skin was marked to get the shortest needle entry route meanwhile avoiding the inclusion of bullae and vessels structures. Embolization microcoil (Cook Incorporated, Bloomington, IN 47404, USA) was selected as a localization marker with a wire diameter of 0.018 inches and a length of 7cm. After local anaesthesia with 2% lidocaine, an 18G/10cm percutaneous introducer needle (Cook Incorporated, Bloomington, IN 47404, USA) was then advanced to puncture from the marked point on the skin without penetrating the parietal pleura. After confirming the direction of the tip of the puncture needle by the second CT scan, further insertion into the normal lung parenchyma around the lesions (within 5mm) was carried out. Then the third CT scan was performed to confirm the final position of the tip of the needle before connecting the loading cannula of the microcoil to the needle. Our method for deploying the microcoil was a modified procedure from Powell’s method [1]. The microcoil was implanted adjacent to nodule within 5mm instead of nodule penetration, which may incite hemorrhage or inflammation in the nodule and then affect histopathologic assessment [2]. The intention of our method was to leave the proximal end of the microcoil on the visceral pleura, which will be a direct clue for nodule’s position during VATS resection and significantly improve the efficiency of surgery [3].

Basing on the learning curve analysis described by Chao et al [4], the over-33-month (from June 2016 to March 2019) operational process of the interventional radiologists could be divided into two stages: the initial 16-month and the later 17-month. In the initial 16-month, the radiologists were with limited experience for evaluating microcoil dislodgement or migration (localization failure) on CT scans and the microcoil implantation used to be given only once for one nodule, while in the later 17-month, a repeated localization procedure would soon be done if the radiologists predicted the proximal end of the first microcoil was likely to dislodge or detach from the visceral pleura on post-procedural CT images.

[1] Powell TI, Jangra D, Clifton JC, Lara-Guerra H, Church N, English J, et al. Peripherallung nodules: fluoroscopically guided video-assisted thoracoscopicresection after computed tomography-guided localization using platinum microcoils. Ann Surg. 2004;240:481-488.

[2] Bommart S, Bourdin A, Marin G, Berthet JP, Pujol JL, Serre I, et al. Impact of preoperative marking coils on surgical and pathologic management of impalpable lung nodules. Ann Thorac Surg. 2014;97:414-418.

[3] Rostambeigi N, Scanlon P, Flanagan S, Frank N, Talaie R, Andrade R, et al. CT fluoroscopic-guided coil localization of lung nodules prior to video-assisted thoracoscopic surgical resection reduces complications compared to hook wire localization. J Vasc Interv Radiol. 2019;30:453-459.

[4] Chao YK, Fang HY, Wen YW, Hsieh MJ, Wen CT. Intraoperative computed tomography-guided pulmonary tumour localization: a thoracic surgeon's learning curve. Eur J Cardiothorac Surg. 2019;55:421-426.

**Assessment**

**Nodule type**: nodules were classified as solid, part-solid and ground-glass opacity (GGO) according to their density on CT images with a lung window setting (level:-450HU; width: 1300HU). A nodule is defined as GGO when it has increased attenuation relative to lung parenchyma but has not as dense as soft tissue (such as the parenchymal vessels). Part-solid nodule contains some areas with solid attenuation.

**Depth from pleura**: the shortest vertical distance between nodule and pleura.

**Presence of emphysem**a: the emphysema region around the needle insertion pathway.

**Patient position for localization procedure**: prone or supine.

**Pleura-microcoil distance**: the distance calculated along the needle insertion pathway.

**Pleural indentation**: defined as the manifestation of the pleura which is not penetrated by the needle but protruding towards the nodule, resulting in a tent-shaped appearance of the pleura.

**Scapulae-covered sign**: nodule is shadowed by the scapulae.

**Procedure-related complications**: pneumothorax or pulmonary hemorrhage.

**Localization procedure time**: defined as the interval time between the initial CT scan scout film before puncture and last CT scan following completion of localization procedure.

**The time to the operation**: defined as the interval time between the termination of the postprocedural CT scan in the interventional unit and the start of the general anaesthesia in the operating room.
